# Meeting the challenges of wild boar hunting in a modern society: The case of France

**DOI:** 10.1007/s13280-023-01852-1

**Published:** 2023-03-21

**Authors:** Pablo Vajas, Erica Von Essen, Lara Tickle, Marlène Gamelon

**Affiliations:** 1 DECOD (Ecosystem Dynamics and Sustainability), IFREMER, INRAe, Institut-Agro-Agrocampus Ouest, rue de L’île d’Yeu, 44311 Nantes Cedex 3, France; 2 Department of Social Anthropology, Stockholm University, Universitetsvägen 10 B, Socialantropologiska Institutionen, 106 91 Stockholm, Sweden; 3 Faculty of Applied Ecology, Agricultural Sciences and Biotechnology, Inland Norway University of Applied Sciences, Innlandet, Norway; 4 Laboratoire de Biométrie et Biologie Evolutive, UMR 5558, CNRS, Université Lyon 1, Campus de la Doua, Bâtiment Gregor Mendel, 43 Boulevard du 11 novembre 1918, 69622 Villeurbanne, France; 5 Department of Biology, Centre for Biodiversity Dynamics, Norwegian University of Science and Technology, Trondheim, Norway

**Keywords:** Human–wildlife conflicts, Hunting laws, Optimal harvesting, *Sus scrofa*, Transdisciplinary perspectives

## Abstract

Modern hunting is an ambivalent practice, torn between leisure and labor. Nowhere are these conflicting dimensions better manifested than for wild boar—a simultaneous game and pest species in many countries. Here, we consider the sociological, political and cultural phenomenon of wild boar hunting from a change perspective, starting at its historical roots to future implications concerning the changing demographics, drivers, needs and practices of a modernizing hunting community. Using the case context of France, we present an approach to deconstructing each component of wild boar hunting firstly, and subsequently the external forces that change the nature of hunting. The objective of this manuscript is to discuss of the wild boar optimal harvesting to be applied in changing social and ecological environment. Findings show that the challenges facing wild boar management will likely intensify in the future, especially under the spotlight of a controversial public debate.

**Supplementary Information:**

The online version contains supplementary material available at 10.1007/s13280-023-01852-1.

## Introduction

For the successful implementation of wildlife management plans, the local involvement of different stakeholders is essential. Managers have to deal with the divergent interests of different stakeholders, especially if changes in some cultural activities are needed, e.g., in hunting-management (Conover 2001; Tombre et al. 2021). In this regard, hunting is an ambivalent activity that has both leisure and labor dimensions (von Essen and Tickle 2020). New legislation and evolution of ethical values in society confront hunters and managers to new constraints of managing wildlife, raising the issue of the operational feasibility of management recommendations (von Essen et al. 2019). Wild boar (*Sus scrofa*) hunting clearly illustrates the ambivalent position of hunting. On the one hand, wild boar’s representation has changed from mystical beast with which to engage in a fierce fight, to feral pigs, or semi-domesticated wild (often named *cochon-glier* or *sangli-chon* from *sanglier*-wild boar- and *cochon*-pig; Gigounoux 2017; von Essen 2019). On the other hand, for some people wild boar represents a “public enemy” to be killed (e.g., farmers), but for hunters they represent the emblematic species that promotes hunting activities. (Mounet 2012; Keuling et al. 2016). As a result, these populations have undergone favorable management measures to “keep” the interest around hunting (Maillard et al. 2010).

Wild boar (*Sus scrofa*) species exhibits an unusual life history among ungulates (Focardi et al. 2008), with high reproductive output early in life from their first year of life (on average 5 piglets per litter and up to 13, see Bieber and Ruf 2005 review), and extraordinary plasticity to environmental conditions (Frauendorf et al. 2016). Under intense hunting pressure, wild boar can adjust their behavior (home range enlargement, nocturnal characteristic reinforcement, see Keuling et al. 2008; Podgórski et al. 2013; Keuling and Massei 2021) and give birth earlier in the season allowing females to reach the reproductive mass threshold in their first year of life (Gamelon et al. 2012). Thus, wild boar management faces the challenge of a species characterized with fast turnover similar to small passerines or rodents (Gaillard et al. 1989). These biological characteristics, as well as environmental changes such as milder winters and changing landscape structure (Vetter et al. 2015; Markov et al. 2019), including expanding agricultural landscapes (agroecosystem; Morelle et al. 2016; Fattebert et al. 2017), as well as artificial feeding (Geisser and Reyer 2004; Cellina 2008) might have contributed to its rapid expansion and distribution across Europe in recent years (Massei et al. 2015; Linnell et al. 2020), now populating even to urban areas (Cahill et al. 2012; González-Crespo et al. 2018). This increase is accompanied by crop and forest damage (Schley et al. 2008; Amici et al. 2012; Burrascano et al. 2015), risk of increased car accidents (Langbein et al. 2011; Thurfjell et al. 2015), impacts on biodiversity (Bruinderink and Hazebroek 1996; Bueno et al. 2013) and increased risks of disease transmission such as African Swine Fever, salmonella or trichinosis (Gortázar et al. 2006; Podgórski and Śmietanka 2018).

How equipped, willing, and capable are modern hunters to meet the challenges of a burgeoning wild boar population, and consequent management objectives to ‘cull’ and mitigate wild boar impacts? This is the central question we address in our manuscript. Because both leisure and labor dimensions underlie modern hunting, and wild boar hunting manifests this clearly, optimal efficiency in terms of maximum harvest is not necessarily sought by all hunters (Keuling et al. 2016; von Essen and Tickle 2020). At the same time, they are under much pressure from other stakeholders to keep numbers down (Mounet 2012; Keuling et al. 2016). Today’s hunters are also changing, in terms of their desires, profiles, practices and affinity to local communities (Ueda et al. 2010; Keuling et al. 2016, 2021). Given this, what is needed is a consideration of the various levels of effectiveness of wild boar optimal harvesting in relation to the constantly varying social and ecological environments. In this sense, understanding the hunting process is therefore of prime importance.

In the hunting process, hunting effort (set of labors implemented to hunt, Vajas et al. 2020) is one of the central measures for controlling wild boar numbers (Apollonio et al. 2010; Keuling et al. 2013). The hunting effort metric often used for wild boar management is the number of "hunter-days", which then has two parameters: the days hunted and the number of available hunters (Vajas et al. 2020, 2021). However, hunting effort is a complex concept since the relationship between effort and harvest (expected objectives of the number of individuals to be shot) is neither linear nor proportional (Bishir and Lancia 1996; Walters 2003). This complex relationship between effort and harvest success is particularly true in recreational fishing or hunting, a context that is characterized by a high effort for a low success (Hilborn 1985; Milner-Gulland et al. 2009). The reason why effort does not equal harvest success can be explained by a key parameter, the catchability, i.e., the probability of an animal to be caught per unit of effort (Arreguin-Sánchez 1996). This has a biological component (e.g., vulnerability of individuals) and a human component (e.g., hunting weather conditions and hunters’ skills; Hilborn et al. 1992; Gascuel 1993). For example, in the case of wild boar hunting, anti-predator behaviors (escape, hiding Keuling and Massei 2021) can change the catchability over time (Thurfjell et al. 2017; Fattebert et al. 2019), and the ability to hunt or the choice of a hunting method also influence the catchability (Keuling et al. 2021; Bergqvist 2022; as in fisheries Marchal et al. 2006). Therefore, the harvest does not depend exclusively on hunting effort invested, and simply increasing hunting effort is unlikely to meet management quotas.

**Fig. 1:**
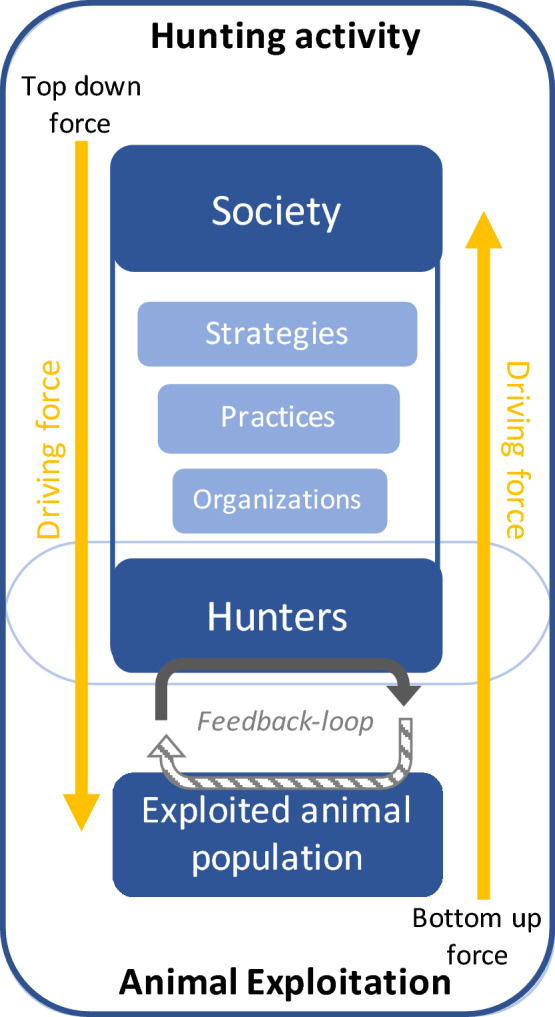
Simplified representation of the forces that drive hunting activity. The “hunting activity” block consists of a “society” made up of all the components of hunting activity, i.e., strategy, hunting practice, and hunting organization, and a “hunters” block influenced by these components. The “hunters” meet the “exploited population” in the “animal exploitation” block. This process is dynamic and will create feedback loops of mutual adaptation to changes in hunting effort and animal population abundance. This whole process can be driven from above, i.e., society, by “top down”, or from below, i.e., the exploited population and the hunters, by “bottom-up”

Studies within ecology increasingly ask the pressing question, in light of the proliferation of wild boar numbers: “what is the most efficient way to decrease wild boar population size or mitigate the nuisances caused?” or “What is the ability of populations to ‘bounce back’ after being harvested”? (see for example Servanty et al. 2011; Gamelon et al. 2012). Meanwhile, the social sciences and humanities, which are in something of a “wild boar turn”, can now be seen engaging more with questions like: “why do we hunt wild boar the way we do?” in regard to norms, practices and ideas about wild boar hunting in modern society (see, for example, von Essen et al. 2019; von Essen 2019). The combination of the two questions, that is, both efficiency and the capacity for extant cultural hunting practices to meet this, raises the question: “What does our current and future way of hunting wild boar mean for both wild boar and hunters?” and, in an ecologies-inspired approach, “how do they affect each other?” We clarify how top-down and bottom-up understandings of the various processes that structure wild boar hunting today allows for a better understanding of the *whole* hunting process (Fig. 1). We decompose hunting into measurable and manageable components as hunting strategy, hunting practices, hunting organization, hunters’ profiles and hunter’s motivations (based on the fisheries framework; Marchal et al. 2006; Marchal 2010). We use France as an example of contemporary hunting culture illustrating what is happening in Western Europe (von Essen et al. 2019), both with regard to wild boar expansion and to changes affecting hunting. To further contextualize and understand future trajectories, we provide an historical overview of hunting laws in France, from the French Revolution to "ecological hunting" as part of the "greening process” nowadays. This framework allows us to explore the complexity of current hunting-related management proposals in the public debate, and some perspectives to meet management objectives.

## Historical overview of hunting laws in contemporary era in France

**Fig. 2:**
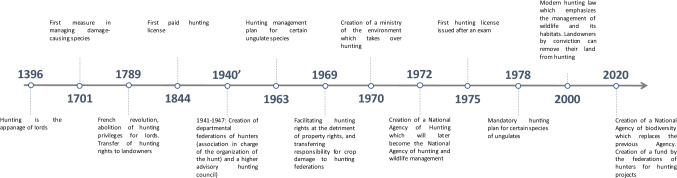
Timeline summarizing the main steps in the historical development of hunting in France

The evolution of hunting laws in France, congruent with the evolution of representations of hunting in society, is essential for understanding the issues around hunting policies today. A timeline is proposed in Fig. 2. From Clovis (481–511) to Louis XVI (1774–1789), 246 laws, edicts and ordinances were written (Estève 2004). Hunting went from being a right that lords could rent on their lands (1500) to being an exclusive right of royalty (Estève 2004). Thus, until the eve of the French Revolution, hunting was an honorary right granted by the king to the nobility and the high justice, intended for distinction and bodily maintenance, and could not be transferred or generate profits (Estève 2004). We can note that during this period, management actions were put in place to regulate the animals causing crop damage. During the French Revolution, two visions opposed each other. On the one hand, Mirabeau's linking the right to hunt and property and, on the other hand, Robespierre's advocating the freedom to hunt for all and in all places for the benefit of all citizens and without conditions (Domas-Descos 2012). Finally, the French Revolution put an end to the seigneurial privilege and everyone had the right to hunt, although this right applied to property rights. From then, it is possible to hunt on these lands, or on the lands of a consenting owner (Estève 2004). In the case of land owned by municipalities, they had the right to rent it from 1804 onwards to tenderers (which opened the door to land rentals from private owners).

During the mid-nineteenth century, many debates reinforced the right of ownership, and excluded the tenants of the woodland from hunting, i.e., the game species, although res nullius was not part of the usufruct (Estève 2004). It was also the period during which large wooded areas were purchased to organize hunting business (Estève 2004). It was aimed to the elite (bankers, merchants, magistrates and lawyers) and caused inflation in the price per hectare of woodland, particularly those close to large towns (or in areas connected by rail). Renting land for hunting was then more expensive than farming, and made up for the costs of crop damage, to the annoyance of the tenant farmers (Estève 2004). In order to limit poaching on their lands, these large landowners progressively set up guarded hunting grounds, increasing tensions around the hunting territories (Estève 2004). We can see here an attempt to restore a past seigneurial practice (from a royal abuse to a bourgeois abuse, Estève 2004). It was then a political issue that crystallized many power relations on different visions in society (capitalism vs. socialism). These great hunts were denounced at the end of the nineteenth century, and debates took place on the nationalization of hunting (Swiss system) or the management of hunting at a more local scale (municipalities, Austro-German system). This was rejected by the opposition, which saw in it an attempt to establish a socialist system and invoked the French Revolution as an argument of authority (Estève 2004).

The most important reform of property rights is the Verdeille law of 1964, after a decrease in the abundance of game species and the number of hunters. It allowed hunters to hunt on a piece of territory belonging to a local hunting association (ACCA) without the owner's consent (forced membership). At this scale, wildlife management strategies were set up (notion of hunting-management) by the departmental hunters' federations (created under Vichy government), giving hunters the freedom to organize hunting as they wished (Estève 2004; Domas-Descos 2012). Linked to the evolution of society with the emergence of ecology and nature protection, in the 1970’s, the Ministry of the Environment was created as well as the French National Agency for Hunting (ONC, which became later French National Agency for Wildlife and Hunting ONCFS, and that is now French National Agency for Biodiversity OFB), and the hunting license could be obtained through an exam (Vigreux 2008). This new representation of hunting has allowed the emergence of new discourses, such as the sporty aspect of hunting as part of nature activities like joggers and cyclists, allowing a clear distinction with the traditional view of "hunting-management" or "bourgeois-hunt" (Fabiani 1982). Thus, the representations that hunting evokes have started to change (Fabiani 1982). In the lobbyist evolution, we can note that in the 1990’s, hunters built a political party, “Chasse, Pêche, Nature et Traditions” (Hunting, Fishing, Nature and Traditions, Vigreux 2008). Playing on the founding myth of the French Revolution, in a sometimes-fantasized folklore, the hunters' political party contrasts cities vs. countryside, rural people (including farmers) vs. urban ecologists, locals vs. distant European officials, acting as defenders of the rural world (Vigreux 2008). This is a unique example of a hobby becoming a political party. In 1999, the European Court of Human Rights sanctioned the mechanism of the Verdeille law, stating that the forced contribution of hunting rights to hunters' associations constituted an interference in the enjoyment of the right to use property (Domas-Descos 2012). In 2000, the law changed and evolved in line with the European Union.

Compared to the Verdeille Law, Law 2000–698 of July 2000 on hunting modifies the rural code. It recognizes the importance of the departmental hunters' federations and the ACCAs (distribution of associative status in France, see Appendix S1 Table S1), but the newness is the possibility of revoking hunting rights on the domain for personal convictions, or revoking them for personal tenancy, but in these cases the owner is responsible for destroying "pests" (Vigreux 2008; Domas-Descos 2012). These laws are part of a national biodiversity strategy (Maillard et al. 2010). They enabled hunting to become more ecological, via a “greening process” of hunting (Alphandéry and Fortier 2007). "Game species" and "territories" progressively become "wildlife" and "habitat". In this framework, a reconfiguration of a system of actors around a common problem was generated: wildlife conservation (i.e., all species, not just protected ones; Fortier and Alphandéry 2012; Ginelli 2012) is accompanied by institutional arrangements seeking to establish consultation plans in favor of a forest—agriculture—wildlife balance (i.e. management policy; Mormont 1996; Maillard et al. 2010; Fortier and Alphandéry 2012). Hunters play the role of operational managers of exploited species in their departments. They have a mixed associative status recognized as “public utility”, i.e., having sufficient funds to be sustainable, and carrying out an activity of general interest. Thus, they must present their management objectives over a period of 6 years, validated by the institution. Departmental federations are legally responsible for damages caused by wildlife and must pay for them from their own funds (such as deer damage to forest seedlings or wild boar damage to crops, Maillard et al. 2010; Domas-Descos 2012). In private forests with fenced areas, hunting is allowed all the year round (law L.424 3); but this is debated, with an ethical evolution in public opinion (Baltzinger et al. 2016). A draught law proposes the prohibition of hunting enclosures on animal ethics grounds (Number 4171, proposals for a law on the prohibition of the enclosure of wild animals for hunting purposes).

## Hunting wild boar: How does it work? Different processes involved

Hunting takes place within a socioecological system, involving complex interactions, uncertainties and feedback loops, between the resource system (ecosystem), the resource unit (e.g., the exploited animal) and society, such as systems of governance (the laws of a society) and their users (e.g., hunters; Redman et al. 2004; Liu et al. 2007; Ostrom 2009). This complexity is aptly illustrated by the case of wild boar hunting. The mere presence of wild boar leads to a cascade of administrative, geographical and management policy consequences (Mounet 2012; Bondon et al. 2021). Hunting, as an activity closely linked to the presence of wild boar, is also influenced by societal ethical standards (von Essen et al. 2019; von Essen and Tickle 2020). To understand the complexity of the hunting process, we separated it into key measurable and identifiable components (Pelletier et al. 2009; Rounsevell et al. 2021). We have defined these components from top down to bottom-up effect namely, hunting strategy, hunting practice, hunting organization, hunters' profiles and individual effectiveness. A simplified representation is provided in Fig. 1.

### Hunting strategy

French hunting is governed by a general strategy driven by an exploited species management policy set at the national level. Allowing a species to be harvested are decisions taken by legislators over a long period of time, with a potential monetary investment including cost–benefit tradeoff from societal sectors and industries who are impacted both positively and negatively by a species presence (i.e., fisheries Marchal et al. 2006; or wild boar Carnis and Facchini 2012). Putman et al. (2010) review socio-legal administrative management clusters for Europe, noting how in France, a model allocates hunting rights to the landowner but retains rights of the state to determine what and how many of the species in question may be culled. Among several important principles are the ownership schemes for wildlife—*res nullius* or *res communis*—the allocation of hunting rights, and the overall purpose of the management: is it to protect wildlife, if so what wildlife and from whom? Alternatively, is it to generate income and activity for hunters? Is it to protect the larger ecosystem? In France, hunting is a lucrative activity that contributes to the economy in whose interest would arguably be to maintain these profits (2 billion euros in 2014; BIPE 2015). Locally, however, landowners and managers may also decide to invest in non-lethal schemes to protect their interests from wildlife damage, such as fencing off crops. In this respect, Saldaqui (2012) uses an interesting expression, namely the "vocation of the territory", to discuss the compromise between forestry production and hunting interests in the investment orientations for hunting activities.

### Hunting practice

In the same area, for a given species, several hunting practices may coexist (see Appendix S1 Tables S2 and S3). The choice of the method or approach to shoot individuals can be cultural, based on tradition, or can evolve to achieve maximal efficiency (see Appendix S1: Table S4; Braga et al. 2010; Keuling et al. 2021; Bergqvist 2022). For example, in Europe several main practices are preferred depending on the country, such as solitary hunting on a stalking post as in Germany or in the Nordic countries (still hunting, pirsch; Keuling et al. 2021; Bergqvist 2022), and collective hunting in the Latin countries as drive hunting as is the case in France (Scillitani et al. 2010; Vajas et al. 2020). This is a hunting which consists of flushing out animals using dogs, with stationary posted hunters who can then shoot them (Vajas 2020; Vajas et al. 2020). Drive hunting involve firearms, while some individual hunts, such as approach or stalking, can be performed with edged weapons such as a bow, where this is legal (von Essen 2019). However, these different techniques are not equivalent in terms of efficiency (number of animals culled per unit of effort) and some practices would seem to be highly effort-consuming in view of the return on investment (see discussion part).

### Hunting organization

Hunting organization can be defined as the sequence of decisions taken by hunters during the hunt (Marchal et al. 2006, 2007). In other words, it corresponds to the means by which hunters allocate their effort in that territory based on a priori knowledge of the expected benefit (Hilborn and Ledbetter 1979). Thus, the hunting organization reflects both hunters’ decisions and the results of past decisions (Pelletier and Ferraris 2000). In fisheries, organization is an operative concept used in bio-economic models where fishermen aim to maximize their return on investment by the best spatial and temporal choices (Marchal et al. 2006, 2007; Marchal 2010). Hunting optimization can be reached through a better temporal and spatial organization of the hunt, and how often they occur, that partly borrows from this model (see discussion part; Jensen et al. 2016a, b).

### Hunters’ identities and individual efficiency

Hunters as a group are heterogeneous and characterized by mixes of different motivations and sociocultural backgrounds, driving individual effectiveness to culling (for France see Appendix S1 Table S5). Motivation can be divided into several categories, namely (i) hunting food/subsistence hunting (e.g., Milner-Gulland et al. 2003), (ii) recreational hunting (e.g., Sharp and Wollscheid 2009), (iii) and 'administrative' hunting (e.g., McCann and Garcelon 2008). On a more local scale, in the context of 'western' hunting, groups of hunters are socially differentiated by their adherence to a certain hunting practice and by a similar hunting ethic and vision (sense of belonging to the same, von Essen et al. 2019). Thus, besides hunting skills, the group to which hunters belong, their motivations and their acceptance of management measures will directly influence the harvest (Keuling et al. 2016; Jaebker et al. 2021). For example, "pro-wild boar" hunters with a utilitarian vision of wild boar will invest more in wild boar hunting in terms of the number of hunting days and number of wild boar harvested than an occasional hunter or a "manager" hunter (Connally et al. 2021a, b). Thus, to each group specific management recommendations can be made (Andersen et al. 2014; Connally et al. 2021a). We can note that these processes of identity, integration into a group or objectives, can lead to conflicts. For example, in the case of wild boar hunting in Japan, the conflict is between hunters and trappers, with the latter preferring to hunt alone around agricultural plots (with the aim of reducing crop damage) without investing in hunting societies (of "rifle" hunters), which is then in decline (Knight 2003; Ueda and Kanzaki 2005; Ueda et al. 2010).

## Discussion

### What are the implications of targeting hunting effort to control wild boar numbers?

#### Should *increasingthe number of hunters* be considered to raise the hunting effort?

Theoretically, one way to increase hunting effort is to increase the number of hunters. Locally, this is a strategy that can be applied by hunters' associations by promoting invitation of external hunters, who can represent up to half of the numbers at the end of the hunting season. At the national level, one strategy used by hunters' associations and the government is to make hunting accessible to a wider audience, by promoting access to hunting licenses (Andersen 2015). Access to hunting licenses has been simplified, their cost has been reduced, and their annual validation has gone from a local to a national scale (see Appendix S1: Table S6). Hunters’ associations can even offer free hunting license exams. Thus, after a sharp decrease in the number of hunters these last decades in France, there was a slight increase during the hunting season 2018–2019. However, there are arguments that can be widely debated. Massei et al. (2015) have shown that when the number of hunters decreases (as in France, Italy or Spain), or when it slightly increases (as in Belgium or Germany), the ratio between the number of wild boar culled and the number of hunters decreases, illustrating the decrease in the relative impact of hunting.

#### Should *more hunting days* be considered to increase the hunting effort?

Another way to increase the hunting effort is to increase the number of hunting days. This can theoretically be done either by increasing the number of hunting trips in a week or by extending the hunting season range. Hunting often takes place during three days a week, on Saturday and Sunday and one extra weekday mainly for retired people (40% of hunters, BIPE 2015). Often more days can be invested in hunting activities during the week, for example to prepare the meat or to look for animal traces before hunting parties. Therefore, drive hunting is already a time-consuming hunt and the addition of a weekday does not seem realistic. Extending the hunting season range already occurs in different departments in France. However, while adjusting the duration of the hunting season may be useful in some specific cases to shot more individuals (Madsen et al. 2016), it is not so obvious for wild boar (Vajas et al. 2021). Indeed, in two French departments, previous work has shown that catchability decreased at the end of the season in some areas (Vajas et al. 2021). This decrease in catchability may come from a change in the behavior of wild boar that adopt anti-predator behaviors (escape, hiding, see Thurfjell et al. 2017; Keuling and Massei 2021) in response to the landscape of fear, reducing their accessibility (Tolon et al. 2009; Fattebert et al. 2019). Changes in catchability during the hunting season may also come from hunter fatigue at the end of the season, as hunting can already represent between 50 and 70 hunting days per hunter per season (Vajas 2020).

#### Should *alternative hunting practices* be considered to increase the hunting effort?

In France, the hunting practice that culls the highest number of wild boars is probably drive hunting (Maillard et al. 2010). This can be explained by the high investment of hunters (in hunters-days) in the drive hunting practice (representing tens of thousands of hunter-days per department, Vajas et al. 2021). But it is possible that the ratio between hunting effort and harvest is higher for other practices (see Elliger et al. 2001; Liebl et al. 2005; Keuling et al. 2008 for comparison). Previous studies have compared the effect of stalking or drive hunting on the number of wild boars culled and the composition of the hunting bag (Braga et al. 2010; Bergqvist 2022). It appears that solitary hunting practices with point bait allow for a better selection, while at high densities drive hunting seems to be better to cull more individuals (Braga et al. 2010; Keuling et al. 2021; Bergqvist 2022). In addition, it is also possible that stalking or lookout is more effective in the pre-hunting season or in different local contexts such as suburban hunting (Kilpatrick and Lima 1999; Doerr et al. 2001; Williams et al. 2013). It should be noted that the effect on wild boar behavior may differ between hunting practices, and thus change the future success (see review Keuling and Massei 2021). Particular attention should be devoted to the complementarity of hunting practices and catching methods (Keuling et al. 2016; Bergqvist 2022). However, hunting practice depends not only on the exploited species but also on cultural practices and local preferences, and this is a component that may be difficult to control by wildlife managers.

#### Can the same management recommendations be made to *newly recruited hunters* as to experienced ones?

Hunting cultures are open systems, and their practices are changing with human societies (Cartmill 1993; von Essen et al. 2019). As mentioned above, drive hunting requires strong local investment, and new hunters do not necessarily spend so much time there, increasingly being urban residents (in Algeria, urban hunters give up more (Boumendjel et al. 2016). Among these new hunters, it seems important to identify the investment they are willing to make in the area, their preference in terms of hunting practice, their attachment to the local territory and their sense of responsibility in the face of the wild boar issue.

In order to recruit more hunters, easier access to the national hunting license has been put in place (see above). However, this measure could encourage the acquisition of hunting licenses by other hunter profiles, perhaps more occasional hunters, who may prefer commercial hunts in territories around France. These hunters may not feel themselves as part of a local community, unified in the management of wild boar at their level. Baticle (2012) introduced the concept of "hunting localism" as fidelity to the hunting territory, where the hunting territory is very close to the birthplace (from the village itself or from the adjoining village). This hunting localism contributes to the local sense of responsibility, as the new urban-based hunters may feel that wild boar is not their problem (Keuling et al. 2016). Thus, this policy may test the strength of the local membership of the hunters, especially since the recruitment and integration of new hunters into a group relies on the social ties between them (Schorr et al. 2014). Furthermore, the choice of a hunting practice may depend on a feeling of belonging to a certain local hunting culture (von Essen 2019). Drive hunting may no longer correspond to the expectations of new hunters, who may prefer solitary hunts, or even more sporting or archaic hunts, such as the fashion for atavism and the return to bow hunting (von Essen 2019). For example, in Japan, in a context of stricter gun laws, new hunters tend to be trappers. This new population is in conflict with the well-established traditional hunters, who do not share the same objectives (leisure versus damage mitigation) and social aspirations (hunting society versus solitary hunting, Ueda and Kanzaki 2005; Ueda et al. 2010). This last point raises questions the temporal stability of the hunting groups. Indeed, the pressure of meeting the minimum quota for wild boar harvest seems to lead to a loss of motivation among hunters, whose investment represents more of a "war effort" posing moral ethical problems of the hunt to "always kill more". Moreover, the context of financial compensation for crop damage by hunters’ association in France is experienced as punishment, and may push hunters to resign, experiencing control of the boar population as a labor.

#### Could *small adjustments of the hunt* be the way forward?

Regardless of hunting organization tactics, small adjustments could quickly help to improve the efficiency of the hunt and may not be as contingent on meeting the demands of a changing hunting demographic. For example, since 2010, walkie-talkies are allowed in France in the context of big game hunting for safety reasons but also to announce hunting actions, and can help to keep the positioned hunters on the alert. Nightvision and semi-automated rifles have recently been legalized in Sweden due to the increase in wild boar, these technological adjustments are similarly aimed at improving hunting efficiency (Tickle et al. 2022). Watchtower stands and especially portable watchtowers could be promoted (Keuling et al. 2021). Another important point may be the calibration of firearms at least once before the start of the season and at each change of ammunition (each bullet has its own ballistics), and by extension offer regular shooting practice sessions to improve accuracy. In this sense, Keuling et al. (2021) suggests training with professionals, and in the same idea we could imagine mentors for young hunters. Dogs, although their effectiveness is discussed and the level of their involvement should be in wild boar hunts is contested (debated in Vajas et al. 2020), could be trained to be more effective (Dahlgren et al. 2012). Finally, if legislation allows, sometimes adjusting the start time earlier or later may allow animals to be taken during their active period (as for wild boar inactive during the hunting day, Keuling et al. 2008; Vajas et al. 2020).

#### What are the management objectives? *A need for clarification*

**Fig. 3:**
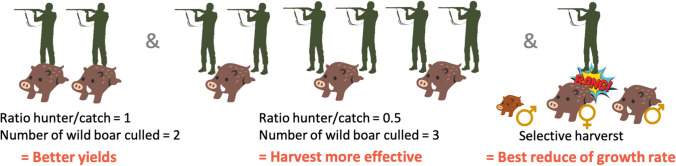
illustration of the different ways of understanding the efficiency of hunting by increasing the ratio of wild boar per hunter, by increasing the hunting bag regardless of the ratio between hunters and wild boar, or by selecting the individuals that have the strongest contribution to population growth rate

Wild boar populations are often judged to be too large. We can then question the need for efficiency in managing wild boar. Is it to reduce the number of individuals at any cost? To maximize the yield between the number of hunters and the number of individuals and thus improve the hunting effort? To reduce local nuisance by hunting or other methods? The implicit objective of several management plans is now to control wild boar population growth rates, not necessarily to kill more individuals. The first way to reach such a goal is to increase the hunting effort to shoot more individuals (Vajas et al. 2020). The second way is to gain efficiency, i.e., to increase the number of individuals culled from the same invested hunting effort (Fig. 3). However, while increasing the number of individuals shot may seem an obvious strategy to control population sizes, the wild boar’s adaptive responses to hunting pressure compensating the loss due to harvest can occur and cannot be ignored (Gamelon et al. 2011, 2021; Keuling et al. 2021). The third way to make wild boar population sizes stable is to shot preferentially individuals that contribute the most to population growth rate. By doing so, the population growth rate can be reduced simply by shooting a low number of individuals (selective harvest, Fig. 3). Finally, local nuisances can be an integral part of management decisions, for example some hunter’s federations have developed a decision table (damage in the cities, in the fields, abundance of people and forest mast) to identify the place and moment for which a greater investment of effort is needed (insert number 3 pages 43–44, Vajas 2020).

## Perspectives

### Towards optimal harvesting: A need for a better understanding of wild boar dynamics

Beyond management objectives aiming efficiency, managers must also deal with all the stakeholders in these local communities, and sometimes beyond. In France, public opinion has recently become critical towards hunting. A recent survey (Ifop 2021, *n* > 1000) has shown that public opinion is increasingly opposed to hunting, due to a feeling of insecurity during hikes (71%), a support for the status of sensitive wild animal (77%), a desire to ban hunting on the Sunday (78% in 2021 against 54% in 2009) and to reduce the hunting season (69%). Spatial and temporal limitations of hunting activity may arise in the future, which may affect hunting effort. For example, the creation of restriction zones, through for example no-shooting policies or through establishing nature reserves, could create reservoirs of wild boar, or the ban of hunting on Sundays might not allow a transfer of hunting effort to other days (Tolon et al. 2012; Vajas et al. 2021).

One potential solution to reconcile hunters and other users of nature is to optimize hunting. For instance, previous work on goose (*Anser brachyrhynchus*) has shown that a better selection of the hunting areas (i.e., the habitats most likely occupied by animals), as well as a better selection of the time periods (i.e., reduced hunting frequency to minimize disturbance and thus flight behavior) allowed to increase the hunting bag (Jensen et al. 2016a, b). These authors also discuss the optimal hunting frequency on the same hunting site. Indeed, disturbance of the hunt will cause individuals to flee (Madsen 1998). It is therefore preferable to wait for the return of individuals to the hunting site to maximize the harvest (Jensen et al. 2016a, b). In this sense, for wild boar, results from Vajas et al. (2021) show that a better temporal allocation of the hunting effort can be proposed according to the hunting condition (type and roughness of the habitat, moment in the hunting season, etc.), and that increasing the size of hunting areas (e.g., by grouping several scattered hunting plots) improves hunting bags (Vajas et al. 2020). The notion of better allocation of hunting effort according to spatial and temporal decisions, including a reflection on the best frequency of hunting in these territories, merits further investigation in the case of wild boar and requires a detailed understanding of wild boar dynamics in space and time.

Optimization of hunting could also result from a better selection of hunted individuals. In population dynamics, some individuals (e.g., depending on their sex, age, body mass) contribute more than others to the population growth rate (Servanty et al. 2011; Gamelon et al. 2012) and thus targeting specifically these individuals can strongly influence the population growth rate. For example, Gamelon et al. (2012) showed that harvesting could be oriented towards large-sized females (> 50 kg) to efficiently decrease the growth rate of a French wild boar population with a limited number of individuals shot (objective reached by killing 20 larger females only, whereas 88 small-sized females (< 30 kg) would need to be killed to reach the same objective). Similarly, the removal of the lead females (the largest females) can have a significant impact on the structure of the herd and reduce the survival of young wild boar (Kaminski et al. 2005; Vassant et al. 2010). While some hunting practices provide an individual selection (Braga et al. 2010; Keuling et al. 2021; Bergqvist 2022), drive hunting is often characterized by the harvesting of the individual that crosses the shooting line. Indeed, the short shooting window of drive hunting does not always allow time to distinguish the sex of individuals, especially sub-adult individuals. In the context of "all-comers" hunting, when a harvest is not systematically carried out during a drive (Vajas et al. 2021), a wild boar seen is a wild boar shot. In this context, it remains an open question how hunting effort could be used to manage the structure of the final hunting bag (Toigo et al. 2008; Bunnefeld et al. 2009, 2011).

Thus, optimal harvesting, defined as minimization of the hunting pressure with maximal reduction of population growth rate, appears to be an interesting strategy to reach management objectives with a reduced impact on the environment, and thus offers promising perspectives to reconcile the expectations of sharing nature between the various stakeholders.

### Towards a better understanding of hunter dynamics

Quantitative studies should next be sought to obtain not only a good description of the wild boar ecology, but also of the hunters’ socio-demography and its change over time (age pyramid, generational variations) in order to keep up demands for the labor and manpower of wild boar management (see Appendix S1 Table S5). Almost universally, the hunting population is aging (see Appendix S1 Table S7), and investment may depend on age (e.g., physical condition, see Appendix S1 Table S8). It will be interesting, therefore, to identify when and by what means a potential generational switch that could take place and bring together the expectations of new hunters and the means that should be used locally to reach management objectives (Andersen et al. 2014; Andersen 2015). Nevertheless, from the hunter with experience of the field to the eager new recruit, hunting profiles do not necessarily accomplish the same management (Andersen et al. 2010; Kaltenborn et al. 2012). As traditional patriarchal structures within hunting diminish and are replaced by increasingly urban populations with minimal attachment to rural living and agribusiness (Gunnarsdotter 2005; Tickle 2019) an element of uncertainty becomes inherent in the wild boar management process. Such uncertainty will have to be dealt with using new means. Suggestions could range from employing professionals to hunt wild boar (with accompanying consequences see von Essen and Tickle 2020; Keuling et al. 2021), payment incentives, mentor schemes, ‘wild boar days’, campaigns or other management options. In addition, it becomes essential to query all types of hunters about their personal feelings of responsibility towards wild boar and its management (Keuling et al. 2016). The feeling of responsibility might not be shared by a new generation more focused on occasional leisure hunting opportunities wherever these arise, and less bound to allegiance of local territories that bear the brunt of boar damage. From the feeling of having in participate to the "war effort", or that of doing the "dirty work", fatigued hunters may "throw in the towel". A study in Sweden shows that a segment of younger hunters (18–35-year-olds) are learning by hunting wild boar and creating their hunting “reputations” by helping farmers protect their crops (Tickle et al. 2022). In interviews, these young hunters express that they feel pressure and responsibility to manage wild boar populations both efficiently and ethically—objectives which are often at odds (Tickle et al. 2022). Therefore, studies will have to be carried out on what hunters are able to achieve and what is acceptable to all (Liordos et al. 2017).

## Concluding remarks

The wild boar is a ubiquitous species with heuristic properties. Emblematic of hunting, the wild boar reveals the ambivalent relationship that hunters have with the species. Our approach decomposes hunting into several components: strategy, practice, organization and hunter characteristics. This allowed us to better tease out where limiting factors to the hunt are located. In contrast to the ecological research from where we import the approach, we have also considered the sociocultural aspects that influence the effectiveness of a hunt. Indeed, since a hunter is a person with a foot both in society and nature (Cartmill 1993), we have tried to accommodate both of these worlds in this work with the hopes of providing a clearer framework for wildlife management that can be adapted to the situation as it evolves both now and in the future of hunting whose debates will intensify, especially under the light of a controversial public debate.

Keuling et al. (2021) argues that beyond the ability of hunters, it is the willingness to change the system that is the main challenge. Our conceptual approach on the hunting process emphasized the top-down (strategy) and bottom-up (hunters) elements that drive management. In this framework, hunters’ motivation and skill can be a consequence of a management strategy as an element driving changes in practices, but also a strong identity element in reaction to the system (Vigreux 2008; Baticle 2012; Jaebker et al. 2021). Elsewhere, we showed how an increase in the number of wild boar and a concurrent decrease in the number of hunters, now contributes to the wild boar being represented as an animal to be excluded from human territory—a boar *non grata* (Tsunoda and Enari 2020). Equally, the wild boar also forms a means of asserting for certain stakeholders their control over the territory (Bortolamiol et al. 2017). Wild boar is now increasingly a point of contention when setting up nature reserves (Boudon 2021). We have also shown how hunting it, and in what format, location and duration, actively reconfigures the wild boar populations locally, including their spatio-temporal behavior (Keuling and Massei 2021). This, in turn, makes new requirements of continued hunting in the area, calling for efforts to be made to keep up with the shifting wild boar (Tolon et al. 2009) and what might be explored further as its ‘response-ability’ (Haraway 2008). While Haraway (2008) meant this as a concept to promote coexistence across the species, we have demonstrated how response-ability also must refer, more instrumentally for managers seeking to control populations, to meeting and accommodating the wiles and behaviors of exploited species. The wild boar is therefore an animal that has the capacity to make the landscape dynamic in a fundamental sense (Bondon et al. 2021).

Hunters are often the guarantors of coexistence with wild boar, having been called the ‘front line’ in the defence of the countryside and natural resources (Mysterud et al. 2020), having the technical means to manage them. They can also be the appointed managers of these species (as in France). However, Europe is in the grip of an epidemic of African swine fever, one of the means to manage the epidemic is hunting (see for France Petit et al. 2021), finding itself in the position of doing the "dirty work" (Emond et al. 2021). In this context, coexistence may become unpredictable and may be marked by radical changes in atmosphere driven by biosecurity management. In this sense, hunters could no longer be stewards of wild boar, but a stakeholder like any other in a society governed by a bio-governance strategy (Broz et al. 2022). In light of this, our findings have emphasized the new—and sometimes uncomfortable—role for hunters as cleaners to perform their duties in the dark, out-of-sight, and uncomplainingly. We believe that their handling of the wild boar situation, including mitigating any disease outbreaks, can make or break hunters in this role. Is the wild boar now a test for hunting to show its utility, and in a way that is palpable to majority society? If so, how experimental can hunters be with new strategies for managing its populations?

## Supplementary Information

Below is the link to the electronic supplementary material.

Supplementary file1 (PDF 786 KB)

## References

[CR1] Alphandéry P, Fortier A (2007). A new approach to wildlife management in France: Regional guidelines as tools for the conservation of biodiversity. Sociologia Ruralis.

[CR2] Amici A, Serrani F, Rossi CM, Primi R (2012). Increase in crop damage caused by wild boar (*Sus scrofa* L.): the “refuge effect”. Agronomy for Sustainable Development.

[CR3] Andersen, O. 2015. Hunter characteristics and preferences for harvest control rules. *PhD thesis*.

[CR4] Andersen O, Vittersø J, Kaltenborn BP, Bjerke T (2010). Hunting desertion in Norway: Barriers and attitudes toward retention measures. Human Dimensions of Wildlife.

[CR5] Andersen O, Wam HK, Mysterud A, Kaltenborn BP (2014). Applying typology analyses to management issues: Deer harvest and declining hunter numbers. The Journal of Wildlife Management.

[CR6] Apollonio M, Andersen R, Putman R (2010). European ungulates and their management in the 21st century.

[CR7] Arreguin-Sánchez F (1996). Catchability: A key parameter for fish stock assessment. Reviews in Fish Biology and Fisheries.

[CR8] Baltzinger M, Mouche J, Blondet M, Hautdidier B (2016). Political ecology de l’engrillagement forestier privé en Sologne: quels sont les enjeux socioenvironnementaux au cœur du conflit?. Natures Sciences Sociétés.

[CR9] Baticle, C. 2012. Le localisme Cynégétique à l’épreuve du Développement durable. Autochtonie et gestion des territoires dans la Somme. *Économie rurale. Agricultures, alimentations, territoires*. Société Française d’Économie rurale: 152–166.

[CR10] Bergqvist G (2022). Harvest bag composition differs among hunting methods for wild boar in Sweden. European Journal of Wildlife Research.

[CR11] Bieber C, Ruf T (2005). Population dynamics in wild boar *Sus scrofa*: ecology, elasticity of growth rate and implications for the management of pulsed resource consumers. Journal of Applied Ecology.

[CR12] BIPE. 2015. Evaluation de l’impact économique social et environnemental de la chasse française. *Cabinet de Conseil en analyse stratégique et prospective économique*.

[CR13] Bishir J, Lancia RA (1996). On catch-effort methods of estimating animal abundance. Biometrics.

[CR14] Bondon, R., R. Mathevet, C. Mounet, and S. Chamaillé-Jammes. 2021. Passer les limites, rythmer le territoire. Paysage et mobilités du sanglier en Valbonnais (Isère, France). *Géocarrefour* 95. Association des amis de la Revue de Géographie de Lyon.

[CR15] Bortolamiol S, Raymond R, Simon L (2017). Territoires des humains et territoires des animaux: éléments de réflexions pour une géographie animale. Annales De Géographie.

[CR16] Boumendjel FZ, Hajji GEM, Valqui J, Bouslama Z (2016). The hunting trends of wild boar (*Sus scrofa*) hunters in northeastern Algeria. Wildlife Biology in Practice.

[CR17] Braga C, Alexandre N, Fernández-Llario P, Santos P (2010). Wild boar (*Sus scrofa*) harvesting using the espera hunting method: Side effects and management implications. European Journal of Wildlife Research.

[CR18] Broz, L., A. G. Arregui, and K. O’Mahony. 2022. Wild Boar Events and the Veterinarization of Multispecies Coexistence. *Understanding Coexistence with Wildlife*. Frontiers Media SA.

[CR19] Bruinderink GG, Hazebroek E (1996). Wild boar (*Sus scrofa scrofa* L.) rooting and forest regeneration on podzolic soils in the Netherlands. Forest Ecology and Management.

[CR20] Bueno CG, Azorin J, Gómez-Garcia D, Alados CL, Badia D (2013). Occurrence and intensity of wild boar disturbances, effects on the physical and chemical soil properties of alpine grasslands. Plant and Soil.

[CR21] Bunnefeld N, Baines D, Newborn D, Milner-Gulland EJ (2009). Factors affecting unintentional harvesting selectivity in a monomorphic species. Journal of Animal Ecology.

[CR22] Bunnefeld N, Reuman DC, Baines D, Milner-Gulland EJ (2011). Impact of unintentional selective harvesting on the population dynamics of red grouse. Journal of Animal Ecology.

[CR23] Burrascano S, Giarrizzo E, Bonacquisti S, Copiz R, del Vico E, Fagiani S, Mortelliti A, Blasi C (2015). Quantifying *Sus scrofa* rooting effects on the understorey of the deciduous broadleaf forests in Castelporziano Estate (Italy). Rendiconti Lincei.

[CR24] Cahill S, Llimona F, Cabañeros L, Calomardo F (2012). Characteristics of wild boar (*Sus scrofa*) habituation to urban areas in the Collserola Natural Park (Barcelona) and comparison with other locations. Animal Biodiversity and Conservation.

[CR25] Carnis L, Facchini F (2012). Une approche économique des dégâts de gibier. Indemnisation, prix et propriété. Économie Rurale Agricultures, Alimentations, Territoires Société Française D’économie Rurale.

[CR26] Cartmill M (1993). A view to a death in the morning.

[CR124] Cellina, S. 2008. Effects of supplemental feeding on the body condition and reproductive state of wild boar Sus scrofa in Luxembourg.

[CR27] Connally RL, Frank MG, Briers GE, Silvy NJ, Carlisle KM, Tomeček JM (2021). A profile of wild pig hunters in Texas, USA. Human-Wildlife Interactions.

[CR28] Connally RL, Frank MG, Briers GE, Silvy NJ, Carlisle KM, Tomeček JM (2021). Hunter motivations and use of wild pigs in Texas, USA. Human-Wildlife Interactions.

[CR29] Conover MR (2001). Resolving human-wildlife conflicts: The science of wildlife damage management.

[CR30] Dahlgren DK, Elmore RD, Smith DA, Hurt A, Arnett EB, Connelly JW (2012). Use of dogs in wildlife research and management. Wildlife Techniques Manual’.

[CR31] Doerr ML, McAninch JB, Wiggers EP (2001). Comparison of 4 methods to reduce white-tailed deer abundance in an urban community. Wildlife Society Bulletin..

[CR32] Domas-Descos MA (2012). Exercice du droit de chasse et droit de propriété. Économie Rurale Agricultures, Alimentations, Territoires Société Française D’économie Rurale.

[CR33] Elliger A, Linderoth P, Pegel M, Seitler S (2001). Ergebnisse einer landesweiten Befragung zur Schwarzwildbewirtschaftung. WFS-Mitteilungen.

[CR34] Emond P, Bréda C, Denayer D (2021). Doing the “dirty work”: how hunters were enlisted in sanitary rituals and wild boars destruction to fight Belgium’s ASF (African Swine Fever) outbreak. Anthropozoologica.

[CR35] Estève C (2004). Le droit de chasse en France de 1789 à 1914. Histoire Societes Rurales.

[CR36] Fabiani J-L (1982). Quand la chasse populaire devient un sport: La redéfinition sociale d’un loisir traditionnel. Études Rurales.

[CR37] Fattebert J, Morelle K, Jurkiewicz J, Ukalska J, Borkowski J (2019). Safety first: seasonal and diel habitat selection patterns by red deer in a contrasted landscape. Journal of Zoology.

[CR38] Focardi S, Gaillard J-M, Ronchi F, Rossi S (2008). Survival of wild boars in a variable environment: unexpected life-history variation in an unusual ungulate. Journal of Mammalogy.

[CR39] Fortier A, Alphandéry P (2012). Les enjeux d’une gestion durable de la faune sauvage La mise en œuvre des ORGFH en France. Économie Rurale Agricultures, Alimentations, Territoires Société Française D’économie Rurale.

[CR40] Frauendorf M, Gethöffer F, Siebert U, Keuling O (2016). The influence of environmental and physiological factors on the litter size of wild boar (*Sus scrofa*) in an agriculture dominated area in Germany. Science of the Total Environment.

[CR41] Gaillard J-M, Pontier D, Allaine D, Lebreton JD, Trouvilliez J, Clobert J (1989). An analysis of demographic tactics in birds and mammals. Oikos.

[CR42] Gamelon M, Besnard A, Gaillard J-M, Servanty S, Baubet E, Brandt S, Gimenez O (2011). High hunting pressure selects for earlier birth date: wild boar as a case study. Evolution.

[CR43] Gamelon M, Gaillard J-M, Servanty S, Gimenez O, Toïgo C, Baubet E, Klein F, Lebreton J-D (2012). Making use of harvest information to examine alternative management scenarios: a body weight-structured model for wild boar. Journal of Applied Ecology.

[CR44] Gamelon M, Nater CR, Baubet É, Besnard A, Touzot L, Gaillard J, Lebreton J, Gimenez O (2021). Efficient use of harvest data: a size-class-structured integrated population model for exploited populations. Ecography.

[CR45] Gascuel, D. 1993. Efforts et puissances de pêche: redéfinition des concepts et exemple d’application. *Les recherches françaises en evaluation quantitatives et modélisation des resources et des systèmes halieutiques, Colloques et séminaires, Paris, Orstom*. Citeseer: 159–181.

[CR125] Geisser, H., and H.-U. Reyer. 2004. Efficacy of hunting, feeding, and fencing to reduce crop damage by wild boars. *Journal of Wildlife Management* 68: 939–946.

[CR46] Gigounoux, A. 2017a. Le sanglier-Chasses, maïtrise des populations et politiques publiques-Approche comparative depuis les confins du Périgord-Noir, du Quercy et du Haut-Agenais.

[CR47] Ginelli, L. 2012. Chasse-gestion, chasse écologique, chasse durable… Enjeux d’une écologisation. *Économie rurale. Agricultures, alimentations, territoires*. Société Française d’Économie rurale: 38–51.

[CR48] González-Crespo C, Serrano E, Cahill S, Castillo-Contreras R, Cabañeros L, López-Martin JM, Roldán J, Lavin S (2018). Stochastic assessment of management strategies for a Mediterranean peri-urban wild boar population. PLoS ONE.

[CR49] Gortázar C, Acevedo P, Ruiz-Fons F, Vicente J (2006). Disease risks and overabundance of game species. European Journal of Wildlife Research.

[CR50] Gunnarsdotter, Y. 2005. Från arbetsgemenskap till fritidsgemenskap: den svenska landsbygdens omvandling ur Locknevis perspektiv [From a community of work to a community of leisure. The changing process of rural Sweden from the perspective of Locknevi]. Uppsala: Swedish University of Agricultural Sciences.

[CR51] Haraway D (2008). When species meet.

[CR52] Hilborn R (1985). Fleet dynamics and individual variation: Why some people catch more fish than others. Canadian Journal of Fisheries and Aquatic Sciences.

[CR53] Hilborn R, Ledbetter M (1979). Analysis of the British Columbia salmon purse-seine fleet: Dynamics of movement. Journal of the Fisheries Board of Canada.

[CR54] Hilborn R, Walters CJ (1992). Quantitative fisheries stock assessment: Choice, dynamics and uncertainty. Reviews in Fish Biology and Fisheries.

[CR55] IFOP. 2021c. Les français et la chasse. *Institut d’études opinion et marketing en france et à l’international*.

[CR56] Jaebker LM, Teel TL, Bright AD, McLean HE, Tomeček JM, Frank MG, Connally RL, Shwiff SA (2021). Social identity and acceptability of wild pig (*Sus scrofa*) control actions: A case study of Texas hunters. Human Dimensions of Wildlife..

[CR57] Jensen GH, Madsen J, Tombre IM (2016). Hunting migratory geese: is there an optimal practice?. Wildlife Biology.

[CR58] Jensen GH, Pellissier L, Tombre IM, Madsen J (2016). Landscape selection by migratory geese: Implications for hunting organisation. Wildlife Biology..

[CR59] Kaltenborn BP, Andersen O, Vittersø J, Bjerke TK (2012). Attitudes of Norwegian ptarmigan hunters towards hunting goals and harvest regulations: The effects of environmental orientation. Biodiversity and Conservation.

[CR60] Kaminski G, Brandt S, Baubet E, Baudoin C (2005). Life-history patterns in female wild boars (*Sus scrofa*): Mother–daughter postweaning associations. Canadian Journal of Zoology.

[CR61] Keuling O, Baubet E, Duscher A, Ebert C, Fischer C, Monaco A, Podgórski T, Prevot C (2013). Mortality rates of wild boar *Sus scrofa* L. in central Europe. European Journal of Wildlife Research.

[CR62] Keuling O, Massei G (2021). Does hunting affect the behavior of wild pigs?. Human–wildlife Interactions.

[CR63] Keuling O, Stier N, Roth M (2008). How does hunting influence activity and spatial usage in wild boar *Sus scrofa* L.?. European Journal of Wildlife Research.

[CR64] Keuling O, Strauß E, Siebert U (2016). Regulating wild boar populations is “somebody else’s problem”!-Human dimension in wild boar management. Science of the Total Environment.

[CR65] Keuling O, Strauß E, Siebert U (2021). How do hunters hunt wild boar? Survey on wild boar hunting methods in the Federal State of Lower Saxony. Animals.

[CR66] Kilpatrick HJ, Lima KK (1999). Effects of archery hunting on movement and activity of female white-tailed deer in an urban landscape. Wildlife Society Bulletin.

[CR67] Knight J (2003). Waiting for wolves in Japan: An anthropological study of people-wildlife relations.

[CR68] Langbein, J., R. J. Putman, and B. Pokorny. 2011c. Road traffic accidents involving ungulates and available measures for mitigation. *Ungulate Management in Europe: Problems and Practices*. Cambridge University Press: 215–259.

[CR69] Liebl T, Elliger A, Linderoth P (2005). Aufwand und Erfolg der Schwarzwildjagd in einem stadtnahen Gebiet. WFS-Mitteilungen.

[CR70] Linnell JDC, Cretois B, Nilsen EB, Rolandsen CM, Solberg EJ, Veiberg V, Kaczensky P, van Moorter B (2020). The challenges and opportunities of coexisting with wild ungulates in the human-dominated landscapes of Europe’s Anthropocene. Biological Conservation.

[CR71] Liordos V, Kontsiotis VJ, Georgari M, Baltzi K, Baltzi I (2017). Public acceptance of management methods under different human–wildlife conflict scenarios. Science of the Total Environment.

[CR72] Liu J, Dietz T, Carpenter SR, Alberti M, Folke C, Moran E, Pell AN, Deadman P (2007). Complexity of coupled human and natural systems. Science.

[CR73] Madsen J (1998). Experimental refuges for migratory waterfowl in Danish wetlands. II. Tests of hunting disturbance effects. Journal of Applied Ecology.

[CR74] Madsen J, Clausen KK, Christensen TK, Johnson FA (2016). Regulation of the hunting season as a tool for adaptive harvest management—First results for pink-footed geese *Anser brachyrhynchus*. Wildlife Biology.

[CR75] Maillard D, Gaillard JM, Hewison M, Ballon P, Duncan P, Loison A, Toigo C, Baubet E (2010). Ungulates and their management in France. European ungulates and their management in the 21st century.

[CR76] Marchal, P. 2010. Dynamique de la mortalité par pêche et impact sur la gestion des ressources halieutiques.

[CR77] Marchal P, Andersen B, Bromley D, Iriondo A, Mahévas S, Quirijns F, Rackham B, Santurtún M (2006). Improving the definition of fishing effort for important European fleets by accounting for the skipper effect. Canadian Journal of Fisheries and Aquatic Sciences.

[CR78] Marchal P, Poos J-J, Quirijns F (2007). Linkage between fishers’ foraging, market and fish stocks density: examples from some North Sea fisheries. Fisheries Research.

[CR79] Markov N, Pankova N, Morelle K (2019). Where winter rules: Modeling wild boar distribution in its north-eastern range. Science of the Total Environment..

[CR80] Massei G, Kindberg J, Licoppe A, Gačić D, Šprem N, Kamler J, Baubet E, Hohmann U (2015). Wild boar populations up, numbers of hunters down? A review of trends and implications for Europe. Pest Management Science.

[CR81] McCann BE, Garcelon DK (2008). Eradication of feral pigs from Pinnacles National Monument. The Journal of Wildlife Management.

[CR82] Milner-Gulland EJ, Bennett EL (2003). Wild meat: the bigger picture. Trends in Ecology & Evolution.

[CR83] Milner-Gulland EJ, Bunnefeld N, Proaktor G (2009). The science of sustainable hunting. Recreational Hunting, Conservation and Rural Livelihoods Science and Practice.

[CR126] Morelle, K., J. Fattebert, C. Mengal, and P. Lejeune. 2016. Invading or recolonizing? Patterns and drivers of wild boar population expansion into Belgian agroecosystems. *Agriculture, Ecosystems & Environment* 222: 267–275.

[CR84] Mormont M (1996). Agriculture et environnement: pour une sociologie des dispositifs. Économie Rurale.

[CR85] Mounet C (2012). Conflits et reconfigurations socio-spatiales autour du sanglier. Des postures générales aux arrangements locaux. Économie Rurale.

[CR86] Mysterud A, Rivrud IM, Gundersen V, Rolandsen CM, Viljugrein H (2020). The unique spatial ecology of human hunters. Nature Human Behaviour.

[CR87] Ostrom E (2009). A general framework for analyzing sustainability of social-ecological systems. Science.

[CR88] Pelletier D, Ferraris J (2000). A multivariate approach for defining fishing tactics from commercial catch and effort data. Canadian Journal of Fisheries and Aquatic Sciences.

[CR89] Pelletier D, Mahevas S, Drouineau H, Vermard Y, Thebaud O, Guyader O, Poussin B (2009). Evaluation of the bioeconomic sustainability of multi-species multi-fleet fisheries under a wide range of policy options using ISIS-Fish. Ecological Modelling.

[CR90] Petit T, Gritti T, Lhote C, Baubet E, Desvaux S (2021). Bilan des opérations de destruction menées en zone blanche sur la population de sangliers. Faune Sauvage.

[CR91] Podgórski T, Baś G, Jedrzejewska B, Sönnichsen L, Śnieżko S, Jedrzejewski W, Okarma H (2013). Spatiotemporal behavioral plasticity of wild boar (*Sus scrofa*) under contrasting conditions of human pressure: Primeval forest and metropolitan area. Journal of Mammalogy.

[CR92] Podgórski T, Śmietanka K (2018). Do wild boar movements drive the spread of African Swine Fever?. Transboundary and Emerging Diseases.

[CR93] Putman R, Putman R, Apollonio M, Andersen R (2010). A review of the various legal and administrative systems governing management of large herbivores in Europe. Ungulate management in Europe: Problems and practices.

[CR94] Redman CL, Grove JM, Kuby LH (2004). Integrating social science into the long-term ecological research (LTER) network: Social dimensions of ecological change and ecological dimensions of social change. Ecosystems.

[CR95] Rounsevell MDA, Arneth A, Brown C, Cheung WWL, Gimenez O, Holman I, Leadley P, Luján C (2021). Identifying uncertainties in scenarios and models of socio-ecological systems in support of decision-making. One Earth.

[CR96] Saldaqui F (2012). L’importance de l’expertise locale dans la gestion concertée de la faune sauvage. Quels enseignements des guides de chasse de l’Office National des Forêts pour les associations communales de chasse? Économie rurale. Agricultures, alimentations, territoires. Société Française D’économie Rurale.

[CR97] Schley L, Dufrêne M, Krier A, Frantz AC (2008). Patterns of crop damage by wild boar (*Sus scrofa*) in Luxembourg over a 10-year period. European Journal of Wildlife Research.

[CR98] Schorr RA, Lukacs PM, Gude JA (2014). The Montana deer and elk hunting population: The importance of cohort group, license price, and population demographics on hunter retention, recruitment, and population change. The Journal of Wildlife Management.

[CR99] Scillitani L, Monaco A, Toso S (2010). Do intensive drive hunts affect wild boar (*Sus scrofa*) spatial behaviour in Italy? Some evidences and management implications. European Journal of Wildlife Research.

[CR100] Servanty S, Gaillard J-M, Ronchi F, Focardi S, Baubet E, Gimenez O (2011). Influence of harvesting pressure on demographic tactics: implications for wildlife management. Journal of Applied Ecology.

[CR101] Sharp, R., and K.-U. Wollscheid. 2009. An overview of recreational hunting in North America, Europe and Australia. *Recreational hunting, conservation and rural livelihoods*. Wiley Online Library: 25–38.

[CR102] Thurfjell H, Ciuti S, Boyce MS (2017). Learning from the mistakes of others: How female elk (*Cervus elaphus*) adjust behaviour with age to avoid hunters. PLoS ONE.

[CR103] Thurfjell H, Spong G, Olsson M, Ericsson G (2015). Avoidance of high traffic levels results in lower risk of wild boar-vehicle accidents. Landscape and Urban Planning.

[CR104] Tickle L (2019). The practice of hunting as a way to transcend alienation from nature. Journal of Transdisciplinary Environmental Studies.

[CR105] Tickle L, von Essen E, Fischer A (2022). Expanding arenas for learning hunting ethics, their grammars and dilemmas: An examination of young hunters’ enculturation into modern hunting. Sociologia Ruralis.

[CR106] Toïgo C, Servanty S, Gaillard JM, Brandt S, Baubet E (2008). Disentangling natural from hunting mortality in an intensively hunted wild boar population. The Journal of Wildlife Management.

[CR107] Tolon V, Dray S, Loison A, Zeileis A, Fischer C, Baubet E (2009). Responding to spatial and temporal variations in predation risk: Space use of a game species in a changing landscape of fear. Canadian Journal of Zoology.

[CR108] Tolon V, Martin J, Dray S, Loison A, Fischer C, Baubet E (2012). Predator–prey spatial game as a tool to understand the effects of protected areas on harvester–wildlife interactions. Ecological Applications.

[CR109] Tombre IM, Fredriksen F, Jerpstad O, Østnes JE, Eythórsson E (2021). Population control by means of organised hunting effort: Experiences from a voluntary goose hunting arrangement. Ambio.

[CR110] Tsunoda H, Enari H (2020). A strategy for wildlife management in depopulating rural areas of Japan. Conservation Biology.

[CR111] Ueda, G., and N. Kanzaki. 2005. Wild boar hunters profile in Shimane Prefecture, western Japan. *Wildlife Biology in Practice*. Sociedade Portuguesa de Vida Selvagem.

[CR112] Ueda G, Kanzaki N, Koganezawa M (2010). Changes in the structure of the Japanese hunter population from 1965 to 2005. Human Dimensions of Wildlife.

[CR113] Vajas, P. 2020d. Évaluation des facteurs influençant le succès de la chasse pour gérer le sanglier (*Sus scrofa*): comprendre les relations entre l’effort de chasse, la capturabilité et les conditions de chasse. Doctoral thesis. Université de Montpellier II. 10.13140/RG.2.2.13505.48488.

[CR114] Vajas P, Calenge C, Gamelon M, Girard F, Melac O, Chandosne C, Richard E, Said S (2021). Catch-effort model used as a management tool in exploited populations: Wild boar as a case study. Ecological Indicators.

[CR115] Vajas P, Calenge C, Richard E, Fattebert J, Rousset C, Saïd S, Baubet E (2020). Many, large and early: Hunting pressure on wild boar relates to simple metrics of hunting effort. Science of the Total Environment.

[CR116] Vassant J, Brandt S, Nivois É, Baubet É (2010). Le fonctionnement des compagnies des sangliers. Faune Sauvage.

[CR117] Vetter SG, Ruf T, Bieber C, Arnold W (2015). What is a mild winter? Regional differences in within-species responses to climate change. PLoS ONE.

[CR118] Vigreux, J. 2008. Le vote CPNT (1989–2002): la chasse, du loisir à la politisation. *Territoires contemporains*: http--tristan.

[CR119] von Essen E (2019). How Wild Boar hunting is becoming a battleground. Leisure sciences.

[CR120] von Essen E, Tickle L (2020). Leisure or Labour: An Identity Crisis for Modern Hunting?. Sociologia Ruralis.

[CR121] von Essen E, van Heijgen E, Gieser T (2019). Hunting communities of practice: Factors behind the social differentiation of hunters in modernity. Journal of Rural Studies.

[CR122] Walters C (2003). Folly and fantasy in the analysis of spatial catch rate data. Canadian Journal of Fisheries and Aquatic Sciences.

[CR123] Williams SC, Denicola AJ, Almendinger T, Maddock J (2013). Evaluation of organized hunting as a management technique for overabundant white-tailed deer in suburban landscapes. Wildlife Society Bulletin.

