# Correction: Meeting the challenges of wild boar hunting in a modern society: The case of France

**DOI:** 10.1007/s13280-023-01932-2

**Published:** 2023-09-09

**Authors:** Pablo Vajas, Erica Von Essen, Lara Tickle, Marlène Gamelon

**Affiliations:** 1 DECOD (Ecosystem Dynamics and Sustainability), IFREMER, INRAe, Institut-Agro-Agrocampus Ouest, rue de L’île d’Yeu, 44311 Nantes Cedex 3, France; 2 Department of Social Anthropology, Stockholm University, Universitetsvägen 10 B, Socialantropologiska Institutionen, 106 91 Stockholm, Sweden; 3 Faculty of Applied Ecology, Agricultural Sciences and Biotechnology, Inland Norway University of Applied Sciences, Innlandet, Norway; 4 Laboratoire de Biométrie et Biologie Evolutive, UMR 5558, CNRS, Université Lyon 1, Campus de la Doua, Bâtiment Gregor Mendel, 43 Boulevard du 11 novembre 1918, 69622 Villeurbanne, France; 5 Department of Biology, Centre for Biodiversity Dynamics, Norwegian University of Science and Technology, Trondheim, Norway

## Correction to: Ambio (2023) 52:1359-1372 10.1007/s13280-023-01852-1

The article “Meeting the challenges of wild boar hunting in a modern society: The case of France”, written by Pablo Vajas, Erica Von Essen, Lara Tickle and Marlène Gamelon, was originally published Online First without Open Access. After publication in volume 52, issue 8, page 1359–1372, the author decided to opt for Open Choice and to make the article an Open Access publication. Therefore, the copyright of the article has been changed to © The Author(s) 2023 and the article is forthwith distributed under the terms of the Creative Commons Attribution 4.0 International License, which permits use, sharing, adaptation, distribution and reproduction in any medium or format, as long as you give appropriate credit to the original author(s) and the source, provide a link to the Creative Commons licence, and indicate if changes were made. The images or other third party material in this article are included in the article's Creative Commons licence, unless indicated otherwise in a credit line to the material. If material is not included in the article's Creative Commons licence and your intended use is not permitted by statutory regulation or exceeds the permitted use, you will need to obtain permission directly from the copyright holder. To view a copy of this licence, visit http://creativecommons.org/licenses/by/4.0/.

The original article has been corrected.

