# Supplementary material for: Meeting the challenges of wild boar hunting in a modern society: The case of France
Source: Ambio. 2023 Mar 21;52(8):1359–72. doi: 10.1007/s13280-023-01852-1 (PMC10271942; doi:10.1007/s13280-023-01852-1)
Supplement: Supplementary file 1 — Supplementary file1 (PDF 786 KB) [file 13280_2023_1852_MOESM1_ESM.pdf]

Ambio

Electronic Supplementary Material
This supplementary material has not been peer reviewed.

Title: **Meeting the challenges of wild boar hunting in a modern society: the case of France**

Authors: Pablo VAJAS, Erica Von ESSEN, Lara TICKLE, Marlène GAMELON

Appendix S1 : Statistical description of hunting activity in France, based on the outputs of the BIPE study, 2015.

Here we propose for information and illustration in an accessible form, the statistical description on the hunting activity proposed by the BIPE (2015) entitled "Economic, social and environmental impact of the hunting sector". Here you can consult the results: https://chasse.bipe.fr/, and here the methodology: https://chasse.bipe.fr/#/Methodo. For information, the study is based on surveys of hunters (>50,000), hunters' association accounts (>9,000), and hunting-related administration (>500). This statistical description allows us to understand the proportion of different status of the hunting associations, the profile of the hunters, the species hunted, the practices used, the frequency of hunting, the economic impact, etc.

Table S1 Extracted from BIPE (2015), https://chasse.bipe.fr/, percentage of the distribution of the different hunts according to the framework in which they take place, e.g., private domain, public domain, in association or owners.

**Public domains**		**Hunting associations**		**Private hunts**		**Commercial hunting**
10%		48%		36%		6%
**Maritime public domain**	**National forest**	**Communal hunting associations**	**ACCA**	**Private owner's hunts**	**Private hunts by action**	
2%	8%	28%	20%	16%	20%	

Table S2 Extracted from BIPE (2015), https://chasse.bipe.fr/, percentage of the different hunting practices in France, the values in bold are on 100% while the other values are a breakdown of the practice by "shooting", themselves on 100%.

**Different hunting practices**	**Pourcentage**
**Shooting (with a rifle or shotgun)**	**84%**
Hunting ahead	30%
Drive hunting (large or small game species)	31%
Stalking hunting	8%
Hunting on the lookout	12%
Hunting at the hut	3%
Passages hunting (e.g. ducks)	11%
Others	5%
**Traditional hunting (e.g., glue)**	**7%**
**Bow hunting**	**3%**
**Falconry hunting**	**1%**
**Hounding (large, small, & underground)**	**5%**

Table S3 Extracted from BIPE (2015), https://chasse.bipe.fr/, different proportions of species hunted in France (100%)

Different types of game species hunted	Pourcentage
Big game species	31%
Small sedentary game species	32%
Migratory landbirds	20%
Waterfowl	13%
Large mountain game species	3%
Small mountain game species	1%

Table S4 Extracted from BIPE (2015), https://chasse.bipe.fr/, proportion of type of species hunted according to the different age groups of hunters. Note, the total is not 100%, as hunters are not exclusive to one type of species hunted.

Age group (years)	All	[15-24]	[25-44]	[45-64]	65+
Big game species	79,17	84,88	79,28	78,58	78,83
Small sedentary game species	82,29	81,81	83,26	83,00	80,43
Migratory landbirds	54,27	53,86	57,18	55,63	49,94
Waterfowl	36,79	52,90	47,45	34,84	28,36
Large mountain game species	6,85	10,11	8,45	6,61	5,36
Small mountain game species	3,69	5,07	4,31	3,45	3,29

Table S5 Extracted from BIPE (2015), https://chasse.bipe.fr/, socio-professional description of hunters as well as French populations and French males. The "Employment" part is calculated on a proportion of 100%, and for the hunter population, 55% of hunters are employed and 45% are retired, unemployed, students and others.

					Employment					
	**Retired**	**Unemployment**	**Student**	**Other**	**Employee**	**Worker**	**Liberal profession**	**Intermediate occupation**	**Craftsman & merchant**	**Farmer**
Hunter semiprofessional group (%)	40	2	2	1	21	15	39	8	9	8
French socioprofessionnel group (%)					29	22	17	24	6	2
French male semiprofessional group (%)					13	34	19	23	8	3

Table S6 Extracted from BIPE (2015), https://chasse.bipe.fr/, proportion of the different hunting licenses (departmental, national, temporary), according to the different age groups of hunters. Note, the high proportion of national licences for the [15-24] class is explained by the fact that it is offered to young hunters

**Age group (years)**	**[15-24]**	**[25-34]**	**[35-44]**	**[45-54]**	**[55-64]**	**[65-74]**	**≥75**
Departmental licence (%) ; total 85%	72.2	80.2	82.5	84.4	85.8	86.7	92.8
National licence (%) ; total 10%	20.1	10.1	9.3	9.5	9.9	10.1	5.8
Temporary licence (%) ; total 5%	7.6	9.6	8.2	6.1	4.3	3.2	1.5

Table S7 Extracted from BIPE (2015), https://chasse.bipe.fr/, age proportion according to the hunter population, French, and French male adult.

**Age group (years)**	**<18**	**[18-24]**	**[25-34]**	**[35-44]**	**[45-54]**	**[55-64]**	**[65-74]**	**≥75**
French hunter population (%); n = 1.1M	0	5	10	13	19	24	19	10
French population (%) ; n = 64.2M	11	8	12	13	14	13	9	9
French male over 18 years population (%); n = 23.9M	0	11	16	17	18	16	12	10

Table S8 Extracted from BIPE (2015), https://chasse.bipe.fr/, percentage of hunter trip frequencies according to age group

**Age group (years)**	**All**	**[15-24]**	**[25-44]**	**[45-64]**	**65+**
Frequency of hunting trips 1-5 (%)	3,58	3,72	4,44	2,77	4,09
Frequency of hunting trips 6-10 (%)	7,40	6,34	7,49	6,38	9,05
Frequency of hunting trips 11-25 (%)	30,30	22,14	27,90	30,26	33,70
Frequency of hunting trips 26-50 (%)	35,87	35,04	36,43	37,44	33,22
Frequency of hunting trips 50+ (%)	22,85	32,76	23,74	23,15	19,94

Reference

BIPE. 2015. Evaluation de l'impact économique social et environnemental de la chasse française. *Cabinet de Conseil en analyse stratégique et prospective économique*. https://chasse.bipe.fr/
